# Co-occurrence of Gitelman syndrome and turner syndrome: a Case Report and literature review

**DOI:** 10.3389/fgene.2026.1808148

**Published:** 2026-07-10

**Authors:** Youhong Luo, Mingrui Liao, Weili Lu, Maoting Li, Chaohua Deng, Yao Zhou, Dandan Yang, Fei Yang, Dan Xu

**Affiliations:** 1 Department of Endocrinology and Metabolism, The Affiliated Hospital of Southwest Medical University, Luzhou, Sichuan, China; 2 Department of Endocrinology and Metabolism, The People’s Hospital of Leshan, Leshan, Sichuan, China

**Keywords:** autoimmune, endocrine, Gitelman syndrome, metabolic, turner syndrome

## Abstract

**Objective:**

To report a rare case of concurrent Gitelman syndrome (GS) and Turner syndrome (TS) and explore their interplay in driving a complex clinical phenotype.

**Design:**

A single-case report with literature review.

**Methods:**

A 34-year-old woman was evaluated via clinical history, laboratory tests (electrolytes, glucose, thyroid function), and genetic analysis (karyotyping, *SLC12A3* sequencing).

**Results:**

The patient presented with short stature, hypokalemia, and hyperglycemia. Genetic testing confirmed a 45, X/46, XX mosaic karyotype (TS) and compound heterozygous *SLC12A3* mutations (GS). Associated conditions included diabetes mellitus, Hashimoto’s thyroiditis, and hyperlipidemia. Insulin and potassium supplementation achieved short-term stabilization.

**Conclusion:**

The coexistence of GS and TS likely synergistically exacerbated metabolic and electrolyte derangements. This highlights the need for multidisciplinary, personalized long-term management in such overlapping genetic disorders.

## 1 Introduction

Gitelman syndrome (GS) and Turner syndrome (TS) are distinct genetic disorders that independently contribute to complex metabolic and developmental abnormalities. GS is an autosomal recessive renal tubulopathy caused by loss-of-function mutations in the *SLC12A3* gene, leading to impaired sodium-chloride reabsorption. It is clinically characterized by hypokalemic metabolic alkalosis, hypomagnesemia, and hypocalciuria (Anne et al., 2017). Conversely, TS results from complete or partial X-chromosome monosomy, typically presenting with short stature, gonadal dysgenesis, and a predisposition to multisystem comorbidities, including autoimmune thyroiditis and glucose metabolism disorders (Gravholt et al., 2023). While the individual pathophysiological mechanisms of these conditions are well-documented, their co-occurrence is exceptionally rare. The concurrent presence of severe electrolyte disturbances driven by GS and the inherent metabolic risks associated with TS poses significant diagnostic and therapeutic challenges. Here, we report a rare case of genetically confirmed GS overlapping with TS, aiming to explore the compounded clinical phenotype and management strategies for this unique comorbidity.

This case report details the diagnostic journey and multidisciplinary management of a patient with concurrent TS and GS complicated by diabetes mellitus. Beyond the rarity of this triad, we discuss the potential compounding impact of GS-associated electrolyte disturbances on the dysregulation of glucose metabolism inherent to TS. By presenting this unique clinical overlap, we aim to highlight the necessity of comprehensive metabolic screening in such patients and provide practical insights into the therapeutic prioritization for managing these complex, co-occurring genetic disorders.

## 2 Case presentation

This study was approved by the Leshan People’s Hospital Ethics Committee. The patient provided written informed consent for publication of her clinical details and accompanying images.

A 34-year-old female presented to our hospital with a complex medical history spanning 8 years. She had a short stature (146 cm) and reported recurrent episodes of bilateral lower limb weakness starting at age 26. Initial genetic testing performed 5 years prior (at an external institution) identified variants in the *SLC12A3* gene and suspected a large deletion on the X chromosome (chrX: 2,699,992-155,257,641). However, a subsequent comprehensive evaluation conducted at an external institution definitively established her diagnosis.

### 2.1 Genetic confirmation

Chromosomal analysis of a blood sample revealed a mosaic pattern: mos 45, X [14]/46, XX [46], confirming Turner Syndrome (TS). This finding corrected the previous suspicion of a large X-chromosomal deletion; the initial result likely represented a technical limitation or misinterpretation of the mosaic cell line. Furthermore, Sanger sequencing of the *SLC12A3* gene confirmed two heterozygous variants: c.1664C>T (p.Ser555Leu) and c.1196G>A (p.Arg399His). These findings indicate a compound heterozygous state, consistent with autosomal recessive GS. The original G-banded karyotype image and Sanger sequencing chromatograms were obtained from an external institution and could not be retrieved for publication; the genetic diagnoses are therefore supported by the written reports and the clinical phenotype.

### 2.2 Clinical history and metabolic status

The patient’s developmental history included menarche at age 14, followed by scanty and highly irregular menses (occurring every 1-2 months). She had a history of one spontaneous pregnancy resulting in a natural miscarriage. In 2017, she was diagnosed with diabetes mellitus (fasting glucose: 10 mmol/L). Upon current admission, she presented with severe hyperglycemia (18.87 mmol/L). Laboratory investigations (Table 1) revealed uncontrolled diabetes (HbA1c 12.4%), hyperlipidemia (Total Cholesterol: 5.80 mmol/L), and positive urine ketones. Additionally, thyroid function tests indicated Hashimoto’s thyroiditis with elevated Thyroglobulin Antibodies (14.00 IU/mL) and Thyroid Peroxidase Antibodies (385.00 U/mL) (Table 1).

**TABLE 1:** Laboratory findings of the patient at admission and during hospitalization.

Test category	Test item	Result	Normal reference range
Electrolytes and minerals	Potassium (K^+^), mmol/L	**2.09**	3.50–5.30
	Sodium (Na^+^), mmol/L	134.6	137.0–147.0
	Chloride (Cl^−^), mmol/L	97.1	99.0–110.0
	Calcium (Ca^2+^), mmol/L	**2.11**	2.20–2.55
	Magnesium (Mg^2+^), mmol/L	**0.54**	0.79–1.03
	Phosphate (P), mmol/L	0.88	0.81–1.45
Lipid and thyroid	Total cholesterol (TC), mmol/L	**5.8**	<5.17
	Low-density Lipoprotein (LDL), mmol/L	**3.91**	<3.37
	Thyrotropin (TSH), mIU/L	**6.123**	0.55–4.78
	Thyroglobulin antibody (TGAb), IU/mL	**14.00**	<4.5
	Thyroid peroxidase antibody (TPOAb), U/mL	**385.00**	<60
24-h urine electrolytes	Urine volume, L/24 h	1.80	​
	Urine potassium, mmol/24 h	59.00	25–100
	Urine sodium, mmol/24 h	191.34	130–260
	Urine chloride, mmol/24 h	217.80	170–250
	Urine calcium, mmol/24 h	**1.48**	2.5–7.5
	Urine magnesium, mmol/24 h	**8.66**	13.0–42.0
	Urine phosphorus, mmol/24 h	4.97	2.1–8.2
Acid-base and ketones	PH	7.454	7.35–7.45
	Free fatty acids (FFA), mmol/L	**0.86**	0.10–0.77
	β-Hydroxybutyrate (β-HB), mmol/L	**0.71**	0.03–0.30
	Lactate (LAC), mmol/L	**2.7**	0.5–2.2
Pancreatic function	Glycated hemoglobin (HbA1c), %	**12.4**	4.0–6.0
	Fasting C-Peptide, ng/mL	0.87	0.81–3.85
	Glutamic acid decarboxylase antibodies (GADA), IU/mL	<1.00	<10.00
Sex hormones	FSH (mIU/mL)	8.85	Follicular Phase:3.85–8.78 Ovulation Phase:4.54–22.51 Luteal Phase:1.79–5.12 Menopause:16.74–113.5
	LH (mIU/mL)	3.30	Follicular Phase:2.12–10.89 Ovulation Phase:19.8–103.3 Luteal Phase:1.20–12.86 Menopause:10.87–58.64
	E2 (pg/mL)	38.83	Follicular Phase:24–114 Ovulation Phase:80–273 Luteal Phase:20–88
	P (ng/mL)	<0.21	Follicular Phase:0.31–1.52 Luteal Phase:5.16–18.56 Menopause:0.08–0.78
	T (ng/dL)	16.44	10–75
	PRL (ng/mL)	13.72	2.74–26.72

Bolded values indicate results outside the normal reference range. Test Instrument: LABOSPECT008 α-3 and cobase801.

### 2.3 Electrolyte imbalance

Consistent with GS, the patient exhibited persistent hypokalemia (Serum K+: 2.09 mmol/L) and hypomagnesemia (Serum Mg2+: 0.54 mmol/L), which remained refractory to intermittent oral supplementation before admission. Other parameters, including immunoglobulins, urine microalbumin-to-creatinine ratio, fundus examination, bone density, and reproductive hormones (LH, FSH, E2, PRL), were within normal limits or non-contributory to the acute presentation (Table 1).

### 2.4 Diagnosis and management

Based on the clinical phenotype and genetic evidence, the patient was definitively diagnosed with concurrent GS, TS, poorly controlled Diabetes Mellitus, Hashimoto’s Thyroiditis, and Hyperlipidemia. Immediate management involved intensive insulin therapy for glycemic control and aggressive potassium replacement. Following the intervention, her blood glucose levels stabilized, and electrolyte disturbances were corrected. Given the chronic nature of both GS and TS, long-term multidisciplinary follow-up is essential to monitor metabolic stability and prevent systemic complications.

### 2.5 Genetic analysis

Genomic DNA was extracted from peripheral blood. Targeted validation of *SLC12A3* variants (NM_000339.2) was performed via PCR and Sanger sequencing. The *SLC12A3* gene is located on chromosome 16q13 and contains 26 exons. The identified variants were: (1) c.1664C>T (p.Ser555Leu) in exon 13, which is predicted to be located within the 11th transmembrane domain based on current protein topology models; (2) c.1196G>A (p.Arg399His) in exon 9, within an intracellular loop. According to ACMG/AMP guidelines (Sue et al., 2015), the p. Ser555Leu variant has been previously reported in multiple GS patients (PM2+PM3_VeryStrong + PP3+PM1). The p. Arg399His variant is predicted to be deleterious by multiple *in silico* tools and absent from large population databases (PM2+PP3+PM5+PM1). Additionally, conventional G-banding karyotype analysis of cultured peripheral lymphocytes (60 metaphases) revealed mosaicism: mos 45, X[14]/46, XX[46].

## 3 Discussion

### 3.1 Clinical spectrum and endocrine-metabolic comorbidities of turner syndrome

In TS, the most common karyotype is 45, X, accounting for 40%–50% of cases (Gravholt et al., 2023; Sue et al., 2015), followed by mosaicism with a 45, X/46, XX karyotype (15%–25%). Other variants include complex mosaicism (e.g., 45, X/47, XXX or 45, X/46, XX/47, XXX); structural X-chromosome abnormalities such as isochromosome Xq (approximately 10% of cases), partial Xp/Xq deletions, and ring chromosomes (often with associated mosaicism); and Y-chromosomal material in 10%–12% of patients, including 45, X/46, XY mosaicism (Gravholt et al., 2017; Cameron-Pimblett et al., 2017). In our case, the patient demonstrated relatively preserved reproductive function, having experienced natural menarche (albeit with oligomenorrhea) and one spontaneous pregnancy that ended in miscarriage. The spontaneous pregnancy is likely attributable to her mosaic karyotype (45, X/46, XX), which is known to confer a higher likelihood of spontaneous fertility than the classical 45, X monosomy (Bernard et al., 2016; Bryman et al., 2011).

Moving to endocrine comorbidities, TS is commonly associated with endocrine comorbidities. A 2018 meta-analysis revealed a 38.6% prevalence of autoimmune thyroid disease among TS women (95% CI 29.7%–47.6%) (Mohamed et al., 2018), with Hashimoto’s thyroiditis affecting 30%–50% of patients—an incidence that increases with age (Mortensen et al., 2009; El-Mansoury et al., 2005; Bakalov et al., 2012). Thyroid peroxidase antibodies are detectable in 40%–45% of cases (Lee et al., 2023), and hypothyroidism occurs across all karyotypes, including mosaic forms. Mechanistically, the heightened susceptibility to Hashimoto’s thyroiditis in TS is primarily attributed to X-linked gene haploinsufficiency (e.g., FOXP3, PTPN22) impairing regulatory T-cell function, genome-wide DNA hypomethylation disrupting immune tolerance, and estrogen deficiency skewing immunity toward a proinflammatory Th1 phenotype; growth hormone therapy may accelerate the clinical expression of pre-existing autoimmunity (de, 2015; Kanakatti Shankar et al., 2025; Krzyscin et al., 2025).

Turning to metabolic disturbances. TS patients have a significantly higher risk of impaired glucose tolerance (IGT) and type 2 diabetes mellitus (T2DM) than the general population. In adults, IGT occurs in 25%–78% and T2DM in approximately 10% of TS individuals (Hjerrild et al., 2011; Gravholt et al., 2019). The underlying mechanisms include X-chromosome gene dosage defects leading to impaired first-phase insulin secretion, altered body composition (central obesity, reduced lean mass), and estrogen deficiency exacerbating insulin resistance, and growth hormone therapy transiently reducing insulin sensitivity, and occasional autoimmune β-cell destruction (Gravholt et al., 2019; Cameron-Pimblett et al., 2025).

Additionally, approximately 50% of TS patients exhibit hypercholesterolemia, characterized by elevated serum triglycerides and LDL cholesterol compared to both 46, XX women and those with primary ovarian failure (Ross et al., 1995; Krzyścin et al., 2024; Van et al., 2006). This dyslipidemia is driven by X-chromosome haploinsufficiency (affecting lipid-related genes), estrogen deficiency (reducing LDL clearance), central obesity, and insulin resistance; growth hormone therapy may transiently increase lipolysis but does not worsen long-term lipid profile (Gravholt et al., 2019). The patient’s dysregulated glucose-lipid metabolism and thyroid autoimmunity are consistent with these characteristic TS manifestations.

### 3.2 Pathophysiological mechanisms and systemic implications of Gitelman syndrome

Similarly, GS, or familial hypokalemia–hypomagnesemia, is the most common inherited renal tubular disorder, with an estimated prevalence of 1–10 cases per 40,000 individuals. Over 350 distinct *SLC12A3* mutations have been identified in GS patients (Takeu et al., 2015; Vargas-Poussou et al., 2011), most often as compound heterozygous mutations, though some patients harbor a single pathogenic variant. In this case, genetic testing revealed two *SLC12A3* variants: (1) c.1664C>T (p.Ser555Leu), a well-documented pathogenic mutation associated with GS (Riveira-Munoz et al., 2007; Balavoine et al., 2011; Cruz et al., 2001), and (2) c.1196G>A (p.Arg399His), a likely pathogenic variant (Cruz et al., 2001; Berry et al., 2013).

Regarding glucose metabolism, GS is frequently associated with glucose metabolism disorders, with diabetes prevalence rates ranging from 14% to 60% - significantly higher than in the general population (Yua et al., 2017; Blanchard et al., 2019). The pathogenesis involves complex, interrelated mechanisms driven by characteristic electrolyte disturbances. Chronic hypokalemia directly impairs pancreatic β-cell function by disrupting ATP-sensitive K^+^ and L-type Ca^2+^ channels, reducing glucose-stimulated insulin secretion (Ren et al., 2013; He et al., 2018; Tseng et al., 2012). Concurrent hypomagnesemia impedes insulin signaling because magnesium is essential for enzymes such as insulin receptor tyrosine kinase. Magnesium deficiency particularly impairs IRS-1 phosphorylation and GLUT4 translocation, thereby promoting peripheral insulin resistance (Piuri et al., 2021; Guerrero-Romero et al., 2008).

Moreover, metabolic dysregulation is further exacerbated by chronic activation of the renin-angiotensin-aldosterone system (RAAS) secondary to renal potassium wasting. Angiotensin II interferes with insulin receptor signaling and activates proinflammatory pathways that worsen insulin resistance (Davis et al., 2006). Consequently, these pathophysiological interactions create a vicious cycle in which electrolyte disturbances both trigger and amplify diabetic pathophysiology, while diabetes-related complications may aggravate the underlying tubular defects (Xin et al., 2025). Hence, comprehensive electrolyte correction—particularly potassium and magnesium supplementation—may help mitigate diabetic progression in GS patients with glucose intolerance.

With respect to growth, severe electrolyte disturbances have also been linked to growth retardation (Kong et al., 2019). Research indicates that GS is closely associated with growth retardation, with approximately 16% of patients exhibiting short stature (Fujimura et al., 2019), which can present as an initial manifestation in childhood, and with a significantly higher risk in males (Riveira-Munoz et al., 2007). The underlying mechanisms are multifactorial: chronic hypokalemia can lead to growth hormone (GH) resistance and reduced local expression of insulin-like growth factor-1 (IGF-1) in the growth plate, directly impairing longitudinal bone growth (Gil-Peña et al., 2010); some patients have concomitant true GH deficiency; specific “severe” mutations in *SLC12A3* (e.g., splicing mutations or mutations causing loss of intrinsic transport activity) result in near-complete loss of Na-Cl cotransporter (NCC) function, providing the genetic basis for growth failure (Riveira-Munoz et al., 2007). Furthermore, estrogen may have a protective effect on NCC, explaining the gender difference whereby males are more prone to severe phenotypes. Therefore, growth should be routinely monitored in pediatric GS patients, and the GH axis should be actively evaluated if growth does not improve despite optimized electrolyte replacement.

Finally, concerning thyroid autoimmunity, GS patients frequently exhibit thyroid abnormalities, encompassing Graves' disease, Hashimoto’s thyroiditis, and thyroid dysfunction, although the underlying mechanisms remain to be elucidated (Zhou et al., 2018). A notable association has been observed between GS and autoimmune thyroid disease (AITD), particularly within East Asian populations (Koca et al., 2024). A large Japanese cohort study found that approximately 4.3% of genetically confirmed GS patients had thyroid dysfunction (Fujimura et al., 2019). A comprehensive literature review identified over 20 cases of GS co-existing with Graves‘ disease or Hashimoto’s thyroiditis (Zhou et al., 2018; Aoi et al., 2007). Although the precise mechanism remains unclear, several hypotheses may explain this association. First, chronic hypomagnesemia, a hallmark of GS, can disrupt T lymphocyte function and promote oxidative stress, potentially breaking immune tolerance and triggering AITD. Second, sustained hyperreninemia and angiotensin II activation in GS exert pro-inflammatory effects that may contribute to thyroid autoimmunity. Third, it remains possible that specific *SLC12A3* mutations directly predispose to AITD, though this requires further functional validation. Thus, for GS patients presenting with fatigue or metabolic disturbances, regular screening for thyroid function and autoantibodies is warranted.

### 3.3 Potential interplay between turner syndrome and Gitelman syndrome

Having discussed each syndrome separately, we now consider their co-occurrence. This case represents a rare co-occurrence of TS and GS. Although TS arises from X-chromosomal abnormalities and GS from *SLC12A3* mutations, their pathophysiological pathways may intersect. Nevertheless, there is an absence of direct mechanistic evidence that would support a “synergistic” interaction. Consequently, the ensuing discourse is hypothesis-generating in nature.

#### 3.3.1 Metabolic interplay

Both syndromes independently impair glucose metabolism. TS confers an inherent risk of insulin resistance and β-cell dysfunction due to X-chromosome gene dosage defects, altered body composition, and estrogen deficiency (Mortensen et al., 2009; Godsland, 2005). GS promotes hyperglycemia through chronic hypokalemia- and hypomagnesemia-induced impairment of insulin secretion and peripheral insulin sensitivity (Yua et al., 2017). In this patient, the coexistence of both conditions may have compounded the metabolic derangements, potentially predisposing her to early-onset and severe diabetes. Nevertheless, without patient-specific data such as C-peptide dynamics, insulin clamp studies, or serial electrolyte measurements after replacement, this remains speculative.

#### 3.3.2 Electrolyte and growth effects

The characteristic hypokalemia and hypomagnesemia of GS not only contribute to metabolic dysregulation but are also implicated in growth retardation (Fujimura et al., 2019). While short stature is a hallmark of TS, GS-related electrolyte derangements could have exacerbated it. Additionally, TS is frequently associated with congenital renal malformations (Ogawa et al., 2021). Subtle structural renal anomalies may intensify tubular dysfunction in GS, complicating electrolyte management. It is important to note that no formal renal imaging was performed in this case to assess this possibility.

#### 3.3.3 Autoimmune considerations

The autoimmune diathesis characteristic of TS—illustrated here by Hashimoto’s thyroiditis—adds another layer of complexity. In TS, X-chromosome haploinsufficiency affects multiple immune-related genes, including FOXP3 (critical for regulatory T-cell function) and PTPN22, leading to impaired immune tolerance (de, 2015; Kanakatti Shankar et al., 2025; Krzyscin et al., 2025). Emerging evidence also suggests an association between GS and autoimmune thyroid disease (AITD) (Zhou et al., 2018; Koca et al., 2024). A recent case report documented GS with co-existent autoimmune thyroiditis (Koca et al., 2024). Chronic hypomagnesemia, a hallmark of GS, can disrupt T lymphocyte function, promote oxidative stress, and potentially break immune tolerance (Piuri et al., 2021). Sustained hyperreninemia and angiotensin II activation in GS exert pro-inflammatory effects that may further contribute to thyroid autoimmunity. Thus, while GS is not classically considered an autoimmune condition, its metabolic and electrolyte disturbances may indirectly modulate immune homeostasis. However, direct evidence linking GS to AITD pathogenesis is still lacking, and larger cohort studies are needed.

#### 3.3.4 Clinical implications and limitations

Despite the absence of definitive mechanistic proof, this case suggests that TS patients presenting with severe electrolyte imbalances or atypical metabolic features may benefit from screening for coexisting tubular disorders such as GS. Conversely, GS patients with unexplained short stature or autoimmune phenomena warrant consideration of TS, especially in the presence of delayed puberty or infertility. Finally, several limitations should be acknowledged. First, comprehensive genetic subtyping for monogenic diabetes was not performed. Second, the family segregation analysis was lacking. Third, only short-term follow-up data are available. Fourth, the proposed synergistic effects remain speculative. Fifth, the original karyotype image and sequencing chromatograms could not be retrieved; the dual diagnoses rely on written genetic reports and the characteristic clinical-biochemical phenotype. Therefore, future studies with longitudinal monitoring are needed.

## 4 Conclusion

In summary, this report presents an exceptionally rare case of concurrent Gitelman syndrome and Turner syndrome. The patient’s complex phenotype—characterized by severe metabolic derangements, electrolyte disturbances, and autoimmunity—suggests a potential compounding effect between these two disorders. However, direct mechanistic evidence remains lacking, and our conclusions are limited by the absence of extended follow-up and original genetic images. Consequently, this case underscores the importance of multidisciplinary evaluation and clinical awareness of overlapping genetic syndromes. Ultimately, long-term, individualized management focusing on electrolyte homeostasis and metabolic regulation is essential, but optimal strategies await prospective studies with longitudinal monitoring.

## Data Availability

The original contributions presented in the study are included in the article/supplementary material, further inquiries can be directed to the corresponding author.

## References

[B1] Anne B. Detlef B. Davide B. Calò Lorenzo A. Etienne C. Olivier D. (2017). Gitelman syndrome: consensus and guidance from a kidney disease: improving global outcomes (KDIGO) controversies conference. Kidney Int. 91, 24–33. 10.1016/j.kint.2016.09.046 28003083

[B2] Aoi N. Nakayama T. Tahira Y. Haketa A. Yabuki M. Sekiyama T. (2007). Two novel genotypes of the thiazide-sensitive Na-Cl cotransporter (SLC12A3) gene in patients with Gitelman's syndrome. Endocrine 31, 149–153. 10.1007/s12020-007-0024-9 17873326

[B3] Bakalov V. K. Gutin L. Cheng C. M. Zhou J. Sheth P. Shah K. (2012). Autoimmune disorders in women with Turner syndrome and women with karyotypically normal primary ovarian insufficiency. J. Autoimmun. 38, 315–321. 10.1016/j.jaut.2012.01.015 22342295 PMC3358475

[B4] Balavoine A. S. Bataille P. Vanhille P. Azar R. Noël C. Asseman P. (2011). Phenotype-genotype correlation and follow-up in adult patients with hypokalaemia of renal origin suggesting Gitelman syndrome. Eur. J. Endocrinol. 165, 665–673. 10.1530/EJE-11-0224 21753071

[B5] Bernard V. Donadille B. Zenaty D. Courtillot C. Salenave S. Brac de la Perrière A. (2016). Spontaneous fertility and pregnancy outcomes amongst 480 women with Turner syndrome. Hum. Reprod. 31, 782–788. 10.1093/humrep/dew012 26874361

[B6] Berry M. R. Robinson C. Karet Frankl F. E. (2013). Unexpected clinical sequelae of Gitelman syndrome: hypertension in adulthood is common and females have higher potassium requirements. Nephrol. Dial. Transpl. 28, 1533–1542. 10.1093/ndt/gfs600 23328711 PMC3685308

[B7] Blanchard A. Vallet M. Dubourg L. Hureaux M. Allard J. Haymann J. P. (2019). Resistance to insulin in patients with Gitelman syndrome and a subtle intermediate phenotype in heterozygous carriers: a cross-sectional study. J. Am. Soc. Nephrol. 30, 1534–1545. 10.1681/ASN.2019010031 31285285 PMC6683723

[B8] Bryman I. Sylvén L. Berntorp K. Innala E. Bergström I. Hanson C. (2011). Pregnancy rate and outcome in Swedish women with Turner syndrome. Fertil. Steril. 95, 2507–2510. 10.1016/j.fertnstert.2010.12.039 21256486

[B9] Cameron-Pimblett A. La Rosa C. King T. Davies M. C. Conway G. S. (2017). The Turner syndrome life course project: karyotype-Phenotype analyses across the lifespan. Clin. Endocrinol. (Oxf) 87, 532–538. 10.1111/cen.13394 28617979

[B10] Cameron-Pimblett A. La Rosa C. Davies M. C. Suntharalingham J. P. Ishida M. Achermann J. C. (2025). Characterization of turner syndrome-Associated diabetes mellitus. J. Clin. Endocrinol. Metab. 110, 1279–1286. 10.1210/clinem/dgae357 38961758 PMC12012693

[B11] Cruz D. N. Shaer A. J. Bia M. J. Lifton R. P. Simon D. B. Yale Gitelman's and Bartter's Syndrome Collaborative Study Group (2001). Gitelman's syndrome revisited: an evaluation of symptoms and health-related quality of life. Kidney Int. 59, 710–717. 10.1046/j.1523-1755.2001.059002710.x 11168953

[B12] Davis P. A. Pagnin E. Semplicini A. Avogaro A. Calò L. A. (2006). Insulin signaling, glucose metabolism, and the angiotensin II signaling system: studies in bartter's/gitelman's syndromes. Diabetes Care 29, 469–471. 10.2337/diacare.29.02.06.dc05-2048 16443917

[B13] de M. A. B. T. (2015). Turner syndrome and genetic polymorphism: a systematic review. Rev. Paul. Pediatr. 33, 364–371. 10.1016/j.rpped.2014.11.014 25765448 PMC4620965

[B14] El-Mansoury M. Bryman I. Berntorp K. Hanson C. Wilhelmsen L. Landin-Wilhelmsen K. (2005). Hypothyroidism is common in Turner syndrome: results of a five-year follow-up. J. Clin. Endocrinol. Metab. 90, 2131–2135. 10.1210/jc.2004-1262 15623818

[B15] Fujimura J. Nozu K. Yamamura T. Minamikawa S. Nakanishi K. Horinouchi T. (2019). Clinical and genetic characteristics in patients with Gitelman syndrome. Kidney Int. Rep. 4, 119–125. 10.1016/j.ekir.2018.09.015 30596175 PMC6308995

[B16] Gil-Peña H. Mejia N. Alvarez-Garcia O. Loredo V. Santos F. (2010). Longitudinal growth in chronic hypokalemic disorders. Pediatr. Nephrol. 25, 733–737. 10.1007/s00467-009-1330-7 19902272

[B17] Godsland I. F. (2005). Oestrogens and insulin secretion. Diabetologia 48, 2213–2220. 10.1007/s00125-005-1930-0 16193292

[B18] Gravholt C. H. Andersen N. H. Conway G. S. Dekkers O. M. Geffner M. E. Klein K. O. (2017). Clinical practice guidelines for the care of girls and women with Turner syndrome: proceedings from the 2016 Cincinnati international Turner syndrome meeting. Eur. J. Endocrinol. 177, G1–G70. 10.1530/EJE-17-0430 28705803

[B19] Gravholt C. H. Viuff M. H. Brun S. Stochholm K. Andersen N. H. (2019). Turner syndrome: mechanisms and management. Nat. Rev. Endocrinol. 15, 601–614. 10.1038/s41574-019-0224-4 31213699

[B20] Gravholt C. H. Viuff M. Just J. Sandahl K. Brun S. van der Velden J. (2023). The changing face of Turner syndrome. Endocr. Rev. 44, 33–69. 10.1210/endrev/bnac016 35695701

[B21] Guerrero-Romero F. Rascón-Pacheco R. A. Rodríguez-Morán M. de la Peña J. E. Wacher N. (2008). Hypomagnesaemia and risk for metabolic glucose disorders: a 10-year follow-up study. Eur. J. Clin. Invest 38, 389–396. 10.1111/j.1365-2362.2008.01957.x 18489400

[B22] He R. Guo W. J. She F. Miao G. B. Liu F. Xue Y. J. (2018). A rare case of hypokalemia-induced rhabdomyolysis. J. Geriatr. Cardiol. 15, 321–324. 10.11909/j.issn.1671-5411.2018.04.005 29915623 PMC5997613

[B23] Hjerrild B. E. Holst J. J. Juhl C. B. Christiansen J. S. Schmitz O. Gravholt C. H. (2011). Delayed β-cell response and glucose intolerance in young women with Turner syndrome. BMC Endocr. Disord. 11, 6. 10.1186/1472-6823-11-6 21406078 PMC3068952

[B24] Kanakatti Shankar R. Gravholt C. H. Backeljauw P. F. (2025). Evolution of health care in Turner syndrome. Am. J. Med. Genet. C Semin. Med. Genet. 199, e32124. 10.1002/ajmg.c.32124 39610314

[B25] Koca O. Alay M. T. Murt A. Yigin A. K. Seven M. Bavunoglu I. (2024). A novel homozygous SLC12A3 mutation causing Gitelman syndrome with co-existent autoimmune thyroiditis: a case report and review of the literature. Cen. Case Rep. 13, 330–338. 10.1007/s13730-023-00845-z 38308744 PMC11442957

[B26] Kong Y. Xu K. Yuan K. Zhu J. Gu W. Liang L. (2019). Digenetic inheritance of SLC12A3 and CLCNKB genes in a Chinese girl with Gitelman syndrome. BMC Pediatr. 19, 114. 10.1186/s12887-019-1498-3 30999883 PMC6471809

[B27] Krzyścin M. Sowińska-Przepiera E. Gruca-Stryjak K. Soszka-Przepiera E. Syrenicz I. Przepiera A. (2024). Are young people with Turner syndrome who have undergone treatment with growth and sex hormones at higher risk of metabolic syndrome and its complications. Biomedicines 12, 1034. 10.3390/biomedicines12051034 38790996 PMC11118016

[B28] Krzyscin M. Sowińska-Przepiera E. Bumbulienė Ž. Syrenicz A. (2025). Might thyroid function in patients with Turner syndrome have a significant impact on their muscle strength. Int. J. Mol. Sci. 26, 3679. 10.3390/ijms26083679 40332280 PMC12027293

[B29] Lee Y. L. Zaini A. A. Idris A. N. Abdullah R. A. Wong J. S. Hong J. S. (2023). Thyroid autoimmunity and autoimmune thyroid disease in Malaysian girls with Turner syndrome: an understudied population. J. Paediatr. Child. Health 59, 879–884. 10.1111/jpc.16405 37066819

[B30] Mortensen K. H. Cleemann L. Hjerrild B. E. Nexo E. Locht H. Jeppesen E. M. (2009). Increased prevalence of autoimmunity in turner syndrome--influence of age. Clin. Exp. Immunol. 156, 205–210. 10.1111/j.1365-2249.2009.03895.x 19298606 PMC2759466

[B31] Mohamed S. Elkhidir I. Abuzied A. Noureddin A. Ibrahim G. Mahmoud A. (2018). Prevalence of autoimmune thyroid diseases among the Turner syndrome patients: meta-analysis of cross sectional studies. BMC Res. Notes 11, 842. 10.1186/s13104-018-3950-0 30486859 PMC6264051

[B32] Ogawa T. Takizawa F. Mukoyama Y. Ogawa A. Ito J. (2021). Renal morphology and function from childhood to adulthood in Turner syndrome. Clin. Exp. Nephrol. 25, 633–640. 10.1007/s10157-021-02031-w 33616778

[B33] Piuri G. Zocchi M. Della Porta M. Ficara V. Manoni M. Zuccotti G. V. (2021). Magnesium in obesity, metabolic syndrome, and type 2 diabetes. Nutrients 13, 320. 10.3390/nu13020320 33499378 PMC7912442

[B34] Ren H. Qin L. Wang W. Ma J. Zhang W. Shen P. Y. (2013). Abnormal glucose metabolism and insulin sensitivity in Chinese patients with Gitelman syndrome. Am. J. Nephrol. 37, 152–157. 10.1159/000346708 23392128

[B35] Riveira-Munoz E. Chang Q. Godefroid N. Hoenderop J. G. Bindels R. J. Dahan K. (2007). Transcriptional and functional analyses of SLC12A3 mutations: new clues for the pathogenesis of Gitelman syndrome. J. Am. Soc. Nephrol. 18, 1271–1283. 10.1681/ASN.2006101095 17329572

[B36] Ross J. L. Feuillan P. Long L. M. Kowal K. Kushner H. Cutler G. B. Jr (1995). Lipid abnormalities in Turner syndrome. J. Pediatr. 126, 242–245. 10.1016/s0022-3476(95)70551-1 7844670

[B37] Sue R. Nazneen A. Sherri B. David B. Soma D. Julie G. (2015). Standards and guidelines for the interpretation of sequence variants: a joint consensus recommendation of the American college of medical genetics and genomics and the association for molecular pathology. Genet. Med. 17, 405–424. 10.1038/gim.2015.30 25741868 PMC4544753

[B38] Takeuchi Y. Mishima E. Shima H. Akiyama Y. Suzuki C. Suzuki T. (2015). Exonic mutations in the SLC12A3 gene cause exon skipping and premature termination in Gitelman syndrome. J. Am. Soc. Nephrol. 26, 271–279. 10.1681/ASN.2013091013 25060058 PMC4310649

[B39] Tseng M. H. Yang S. S. Hsu Y. J. Fang Y. W. Wu C. J. Tsai J. D. (2012). Genotype, phenotype, and follow-up in Taiwanese patients with salt-losing tubulopathy associated with SLC12A3 mutation. J. Clin. Endocrinol. Metab. 97, E1478–E1482. 10.1210/jc.2012-1707 22679066

[B40] Van P. L. Bakalov V. K. Bondy C. A. (2006). Monosomy for the X-chromosome is associated with an atherogenic lipid profile. J. Clin. Endocrinol. Metab. 91, 2867–2870. 10.1210/jc.2006-0503 16705071

[B41] Vargas-Poussou R. Dahan K. Kahila D. Venisse A. Riveira-Munoz E. Debaix H. (2011). Spectrum of mutations in Gitelman syndrome. J. Am. Soc. Nephrol. 22, 693–703. 10.1681/ASN.2010090907 21415153 PMC3065225

[B42] Xin Y. Yin Y. Zhu L. Wang Y. Wu T. Xu J. (2025). Hypomagnesemia induces impaired glucose metabolism and insulin resistance in patients with Gitelman syndrome. Diabetes Res. Clin. Pract. 223, 112160. 10.1016/j.diabres.2025.112160 40164390

[B43] Yuan T. Jiang L. Chen C. Peng X. Nie M. Li X. (2017). Glucose tolerance and insulin responsiveness in Gitelman syndrome patients. Endocr. Connect. 6, 243–252. 10.1530/EC-17-0014 28432081 PMC5632718

[B44] Zhou H. Liang X. Qing Y. Meng B. Zhou J. Huang S. (2018). Complicated Gitelman syndrome and autoimmune thyroid disease: a case report with a new homozygous mutation in the SLC12A3 gene and literature review. BMC Endocr. Disord. 18, 82. 10.1186/s12902-018-0298-3 30409157 PMC6225570

